# Digital lifelines, data landmines: digital mental health tools for regional and urban families in an age of surveillance

**DOI:** 10.3389/fdgth.2026.1941307

**Published:** 2026-10-07

**Authors:** Isobel Butorac, Roisin McNaney, Joshua Paulo Seguin, Jaimie C. Northam, Marie B H Yap, Adrian Carter

**Affiliations:** 1 Turner Institute, School of Psychological Sciences, Monash University, Melbourne, VIC, Australia; 2 School of Computing and Information Systems, Faculty of Engineering and IT, University of Melbourne, Melbourne, VIC, Australia; 3 School of Computing and Information Systems, Faculty of Engineering and IT, University of Melbourne, Melbourne, VIC, Australia; 4 Faculty of Information Technology, Monash University, Melbourne, VIC, Australia; 5 Action Lab, Faculty of Information Technology, Monash University, Sydney, VIC, Australia; 6 The School of Psychology, The University of Sydney, Melbourne, NSW, Australia; 7 Monash Bioethics Centre School of Philosophical, Historical and Indigenous Studies and the School of Psychological Sciences, Monash University, Melbourne, VIC, Australia

**Keywords:** digital mental health, diverse communities, human computer interaction, qualitative methods, regional communities, user-centered design, web-based and mobile health interventions, young people

## Abstract

**Background:**

Depression, anxiety and behavioural disorders are among the leading causes of illness and disability for young people (aged 16–24 years), with suicide the third leading cause of death for individuals aged 15–29 years. For many young people, parents and caregivers play a central role in initiating help seeking, a dynamic shaped by developmental factors, cultural norms, and systemic barriers to accessing professional services, with most adolescents expressing a preference to rely on parental support rather than engage with mental health professionals. Additional barriers for accessing mental health services can include being too far away from clinicians, stigma, and low mental health literacy. Digital mental health (DMH) tools offer the potential to improve mental health service provision for young people and their parents. However, it is important to consider how the data collected from DMH tools is stored, shared or re-used, particularly given the existing biases in the mental health system and the possible impact this could have on a digitally engaged young person in the future.

**Objective:**

We aimed to understand the barriers to mental health service access for parents and young people living in urban and regional locations in Australia, and how they value DMH data and whom they would share it with. We then compared their experiences to explore any differences and similarities in their understanding of DMH tool development and the possible benefits and burdens of the data that is collected when engaging with these tools. The study was part of a larger body of work that contributed to the design of a national mental health and wellbeing web-based “check-in” in Australia.

**Methods:**

We ran a series of user-centred workshops (urban young people, *n* = 10, urban parents, *n* = 7, regional young people, *n* = 6, and regional parents, *n* = 8, total *n* = 31) that explored the perspectives of regional and metropolitan diverse parents and young people in Australia about engaging with DMH tools. We used a semi-structured activity guide to learn about participant perspectives on data security, access sharing custodianship, and potential data-reuse, as well as design preferences for future DMH tools.

**Results:**

The study found that attitudes toward mental health and how it is discussed can vary, with mental health disclosures depending on the family context and systemic societal influences. Participants were concerned about data privacy and tended to keep their online persona separate or deidentified. Lastly, our study found that young people want a tool that is centralised with their caregivers and service providers while parents wanted a tool that is personalised, targeted, and includes resources on parenting and psycho-education on mental health and neurodiversity.

**Conclusions:**

Our findings revealed that particpants’ social identities were associated with their likelihood of enacting help seeking behaviours. To overcome structural and systemic barriers such as stigma and discrimination, we suggest that DMH tools incorporate pseudonymous or user-controlled personas that are deidentified and confidential to increase safe sharing and trust. We recommend the use of a digital integrated support network that could allow young people and their caregivers, teachers, clinicians and mental health professionals to access mental health support.

## 1 Introduction

### 1.1 Mental health of young people in Australia

In Australia, around 39% of young people experienced high or very high psychological distress (1) and almost two in five (38.8%) young people had a mental health disorder that was persistent for 12 months or longer (2). Yet, even when support is made available, many young people do not access appropriate care (3, 4). Young people who experience mental health challenges can face social exclusion, discrimination and stigma that can undermine their willingness to seek help and increase to possibility of engaging in risk taking behaviours (e.g., substance use for self medication) (5). The early emergence of psychopathology can perpetuate these ongoing challenges across the lifespan, having a broad impact on a range of functional domains. For example, complex mental health issues and poor physical health can be exacerbated by limited social, educational, and economic opportunities (6–8). For some young people, sociodemographic risk factors such as low resource schools, discrimination, greater childhood adversity, and lower access to mental health treatment (9, 10) are often experienced together and can be further compounded by intersecting identities (e.g., ethnic minorities, low socioeconomic status, sexual and gender minorities) that marginalise them in multiple ways (11, 12).

Longstanding structural barriers from research and industry has led to users' needs and preferences being overlooked. Researchers have begun to recognise the important value of human-centred design approaches that question the barriers to care (13, 14). To best inform how mental health service provision could be improved for young people, contrasting the perspectives and experiences of both young people and their parents or caregivers could help to build a better understanding of how to best match digital mental health technologies to their needs. This study aimed understand the barriers to mental health service access for parents and young people living in urban and regional locations in Australia, and how they value digital mental health data and whom they would share it with. Previous research within the same project explored this in culturally diverse (15) and LGBTQ+ (16) communities. Amid increasing datafication (17) individuals, particularly young people, are simultaneously empowered and exposed to risks through digital data. It is therefore critical to build data literacy and ethical engagement with DMH tools. Our study addresses this need by examining how diverse regional and metropolitan parents and young people in Australia understand and navigate DMH data, including whom they trust to share it with and how they perceive its benefits and burdens. Through a series of user-centred workshops we identified key barriers to mental health service access and the sociocultural factors shaping data-sharing attitudes. We found that openness to mental health disclosure varied across family and societal contexts, with participants emphasising privacy, control and contextualised support. The findings also emphasised the importance of the school setting for further DMH design consideration. These findings informed the design of a national digital “check in” platform, highlight how DMH tools can be designed to foster trust through deidentified personas, integrates support networks, and culturally response psycho education. Our contribution advances understanding of how data literacy, intersectionality, and social identity shape engagement with DMH tools, offering practical implications for equitable and ethical digital mental health design. Our work contributes empirical insights into the perspectives of diverse parents and young people from both regional and metropolitan contexts, attending to the intersectional dimensions of participants' experiences. Our study extends the literature by qualitatively exploring the lived experiences of both young people and parents from varying geographical areas, through the use of in-depth qualitative inquiry across diverse social contexts, provides a nuanced, intersectional account of DMH engagement that is hard to achieve in existing quantitative or single-suer design studies. Collectively, these contributions advance understanding of how data literacy, identity, and context can inform the ethical and culturally responsive design of digital mental health interventions.

## 2 Background

### 2.1 Sociocultural barriers to mental health access

Around three quarters (75%) of Australian young people live in major cities, with 1 in 5 (20%) living in the lowest socioeconomic areas (1) and just over 1 in 5 (21%) were born overseas (18). Sociocultural factors such as religion and spirituality, culture values and traditions, socioeconomic status and financial barriers, as well as uncertainty toward the future play a major role in the mental health and wellbeing of young people (19). Barriers to mental health treatment include lack of health insurance, financial constraints, limited provider availability, regional workforce shortages, and logistical issues (i.e., child care, work, and transportation), as well cultural differences in understanding mental health and misalignment of interventions with cultural values (20–22). Young people may not be aware of the services available, have experience negative interactions with adults, or may have experienced fragmented communication between service providers and families (23, 24) that may deter help seeking.

### 2.2 The importance of the parent-child relationship to support mental health seeking

Mental health challenges during childhood and adolescence can undermine important milestones such as identity and relationship formation, completion of education or vocational training, and the development of autonomy (25–27). Parents and caregivers shape young people's understanding and preferences for seeking mental health support, with family members being found to the be most relied upon source of support by young people experiencing mental health challenges (28). However there are some barriers for a young person to disclosing their mental health challenges to those they may trust the most. Embarrassment and concern about a family member's negative reaction can prevent a young person from sharing (28), as well as limited parental mental health literacy (29). Parents are often the primary decision maker in determining whether they seek professional help for their child and remain influential as the young person enters adolescence (30, 31) and limited parental mental health literacy may result in a reluctance or delay in seeking professional support for their children (32). A comprehensive systemic review of family factors associated with service use identified parental burden (33, 34), parent problem perception (35, 36) and parent perception of need (37–42) as determinants on whether a caregiver will seek mental health support for their child (29). Given the importance of this relationship and how it informs future help seeking, parents and young people need to be aware of how they can access the appropriate services, and in return, services must meet the needs of the young people they hope to support.

### 2.3 Could DMH tools bridge the gap to mental health service provision in Australia

Young people are the most digitally connected age group across the lifespan, with seven in ten (71%) young people globally using the internet daily (43). In Australia, young people spend over 14 h a week online (44), and an estimated 91% of young people access the internet or own smartphones (45). As digital technologies continue to rapidly advance and become embedded in daily life, young people have increasingly begun to refer to smartphone apps, websites, and social media to self-manage their mental health (46). Digital mental health tools (DMH tools) offer a scalable solution to improve the quality and accessibility of mental health care (47). DMH tools include both preventative and therapeutic interventions that can be delivered through smartphones (48), or web-based platforms (49) and AI enhanced tools that can predict, diagnose and personalise treatment to individuals (50–52). For example, one study found ChatGPT could effectively diagnose mental health conditions based on standardised questions or via simple case studies (53). Digital phenotyping, or the “moment-by-moment quantifications of the individual-level human phenotyping *in-situ* using data from smartphones and other personal digital devices” p. 2 (39, 40, 50, 54) has been used to predict suicide risk (41), relapse of schizophrenia (42), and the severity of depression (55). The collection of *in-situ* real world data holds potential for continuous and objective data collection for psychiatric symptoms, at a lower cost, with an increased capacity to assess more individuals simultaneously (56).

DMH tools offer a solution to many of the barriers encountered when trying to access traditional mental health support. Specifically, DMH tools can provide access to mental health services for those living in rural communities (57), empower patients through digital connectivity and digital resources (58), improve mental health literacy (59, 60) reduce or remove waiting times, and be provided at a much lower cost. They can also deliver mental health screening and signposting for treatment for users with subthreshold symptoms (61), management and continuous monitoring of acute symptoms to track fluctuations (62, 63) and risk assessments and real time support for users at risk of self-harm (64). These technologies could be particularly useful for those who wish to avoid face-to-face interactions (57, 65) or who live in remote areas where options for access to in-person support are limited (66, 67). Furthermore, parents and caregivers should not be expected to support their child on their own. DMH tools offer the potential to provide parenting programs that parents can access at a time that suits them (68), that could include self-advocacy training and skills development (69, 70). DMH tools could be particularly useful for those who live too far away from mental health clinicians or are concerned about stigma.

Despite the potential advantages, there are barriers to using DMH services. In order for DMH services to work, individuals need to be aware of them, know when to use them, and be able to access them. Those living remotely experience problems in all of these areas – not knowing where or when to use such tools, and often having poor access to stable internet, creating a digital divide that further exacerbates DMH inequities (13, 71). Further, those who have low English and/or mental health literacy are less likely to access digital technologies (8, 72) and there is risk of identity-based bias that may not capture diverse end user's experience (73). More work is needed to understand how to create and promote DMH technologies to help bridge this inequality.

### 2.4 Digital mental health data collection, storage and retention

As the landscape for data-driven healthcare expands, considerations for how data is stored and retained is becoming more urgent. Given the sensitive nature of mental health data, considerations ought to be made on whether there is risk of reidentification (74, 75), whether pre-existing biases may be inbuilt in the design of DMH tools around demographic information or mental health assessments collected (76, 77) and whether an end user can comprehensively consent to how long their data is retained (78). Whilst countries in Europe have begun to tackle the potential future uses of data collection through the “Right to be forgotten” (Article 127) which provides individuals the right to request their personal data is deleted from an organisation (78), the rapid expansion and continuous engagement of the digital landscape means that the responsibility is placed on the individual instead of the creators, governments and institutional bodies. As technology and data collection continues to grow, it will become vital to consider how private agencies and governments communicate where an end user's data is stored, used, or shared. This may require an end user to have a level of data governance literacy. One mode of teasing out the social and ethical implications of data governance is through speculative design approaches that engage different communities in discussions around data collection and governance (79). This raises an important question of whether academics and researchers can avoid inherent biases or assumptions based on the popular Westernised, middle class, white demographics this technology tends to be built for, and instead design for parents and young people from diverse socio-cultural, economic and geographical backgrounds who face additional accessibility barriers. Society is experiencing an age of datafication whereby individuals can be both empowered and harmed by digital data (80). It will be crucial to train young people to critically engage with digital technologies, building data literacy through schooling and training programs (81), community-based programs (82), and even using the DMH tools themselves, to encourage them to consider the possible downstream impacts of data collection and distribution.

### 2.5 The importance of the parent-child relationship to support mental health seeking

Mental health challenges during childhood and adolescence can undermine important milestones such as identity and relationship formation, completion of education or vocational training, and the development of autonomy (25–27). Parents and caregivers shape young people's understanding and preferences for seeking mental health support, with family members being found to the be most relied upon source of support by young people experiencing mental health challenges (28). However there are some barriers for a young person to disclosing their mental health challenges to those they may trust the most. Embarrassment and concern about a family member's negative reaction can prevent a young person from sharing (28), as well as limited parental mental health literacy (29). Parents are often the primary decision maker in determining whether they seek professional help for their child and remain influential as the young person enters adolescence (30, 31). A comprehensive systemic review of family factors associated with service use identified parental burden (33, 34), parent problem perception (35, 36) and parent perception of need (37–42) as determinants on whether a caregiver will seek mental health support for their child (29). Parents often seek insights from the lived experiences of others to guide their parenting; however, this process can be shaped by feelings of judgment and self-doubt (83). Given the importance of this relationship and how it informs future help seeking, parents and young people need to be aware of how they can access the appropriate services, and in return, services must meet the needs of the young people they hope to support.

### 2.6 Considerations for designing DMH tools for regional communities

The coordination of mental health services is an ongoing challenge for regional Australia (84) with fewer mental health professionals working in rural or remote areas compared to major cities (i.e., 90 vs. 15–55 psychologists per 100,000/population) (85). Furthermore, regional mental health services are less likely to have the necessary specialised service pathways available to support young people with complex mental health conditions compared to urban areas (86). However those living in major urban areas also face certain challenges as young people with complex mental health show high levels of isolation, unemployment and poverty (87). Whilst digital inclusion in Australia has increasingly improved since the COVID-19 pandemic as a result of more health services being delivered online (88), research into how to appropriately design DMH tools and services for young people and parents living in regional areas is lacking.

For those living regionally, limited motivation for engagement informed by beliefs, preferences and perceptions regarding mental health treatment, has been identified as a barrier for regional users to engage in DMH tools, as well as expectations that an online treatment or intervention will be ineffective (89). Additionally, rural identity (90) and higher levels of stoicism and self-reliance found in regional areas can impact the threshold for help-seeking (91). For young people living regionally, stigma and shame have previously been identified as barriers to help seeking despite most having access to a smartphone (92). Digital literacy of regional clinicians has been previously explored (93), but consideration for whether digital literacy and generational differences between regional and urban end users and its impact on engagement with DMH tools needs to be further explored. As a result of these cultural norms of self-resilience and limited service infrastructure, there may be heightened levels of scepticism toward DMH tools or interventions. Conversely, users may encounter fragmented services (94) and information overload (95, 96) which could influence expectations for DMH tools that coordinate care. These contextual variations underscore that engagement with DMH tools is shaped not only by individual literacy or motivation but by the socio-geographic environments in which users live. Designing DMH tools that reflect these contextual realities will be crucial to promoting inclusive and effective digital mental health care.

## 3 Methods

### 3.1 Study design

The study was conducted as part of a series of research projects (15, 97) that were designed to inform the design and development of a large-scale national online mental health “check in” as part of the Growing Minds Australia Clinical Trials Network (98). We created an activity guide that was centred on a “digital mental health vault” that was then used as a vessel to describe current and future based scenarios about mental health data, privacy and trust (15). We used user-centred design methods (99, 100) to identify potential issues and preferences of parents and young people if they were to engage in DMH tools using 4 activities (an example of the activity schedule seen in Figure 1 and in Table 1). Our approach compared the experiences of parents and caregivers with young people's perspectives on engaging with DMH tools. We ran a series of workshops in metropolitan Melbourne and regional Victoria with young people (aged 15–24 years) and parents of young people (aged 28–67 years) who identified as being from culturally diverse backgrounds, lived regionally or rurally, and as people with mental illness or were living with a disability. We recruited participants with the help of local multicultural mental health focused organisations in metro and regional locations, with the preference to recruit participants with a basic level of mental health literacy due to the time limited nature of the project. The mental health organisations that assisted with recruitment were focused on advocacy and support for multicultural community members. The participants in the regional locations lived in small townships that were close to central regional town that had a population of around 100,000 people. The inclusion of a regional site allowed for exploration of how differences in service access, infrastructure and community connectedness might shape attitudes toward DMH support, without over extending the comparative claims possible within a small, in-depth qualitative sample. We chose to include young people over the age of 18 as this is a distinct life stage where parental influence can still be significant (i.e., financial support, gaining employment or an occupation, co-residence, pro-social behaviours, and mental health support) and in many cultural groups parents and caregivers remain central to decision-making and help-seeking (101–103) as research has shown that during the transition to adulthood, closeness with and emotional support from parents or caregivers increases (104, 105). We collected optional demographic details in the consent process including whether participants identified as having a disability, mental health history and what cultural background they identified with. Each workshop ran for 2.5–3 h with a 20 min refreshment break. We supplied lunch to create a social atmosphere for each workshop. Our findings were provided to the Growing Minds Australia organisation to assist in their development of a national digital service that would be designed to be more inclusive. The design preferences developed within this series could inform future iterations of DMH tools such as online platforms and smartphone apps.

**Figure 1:**
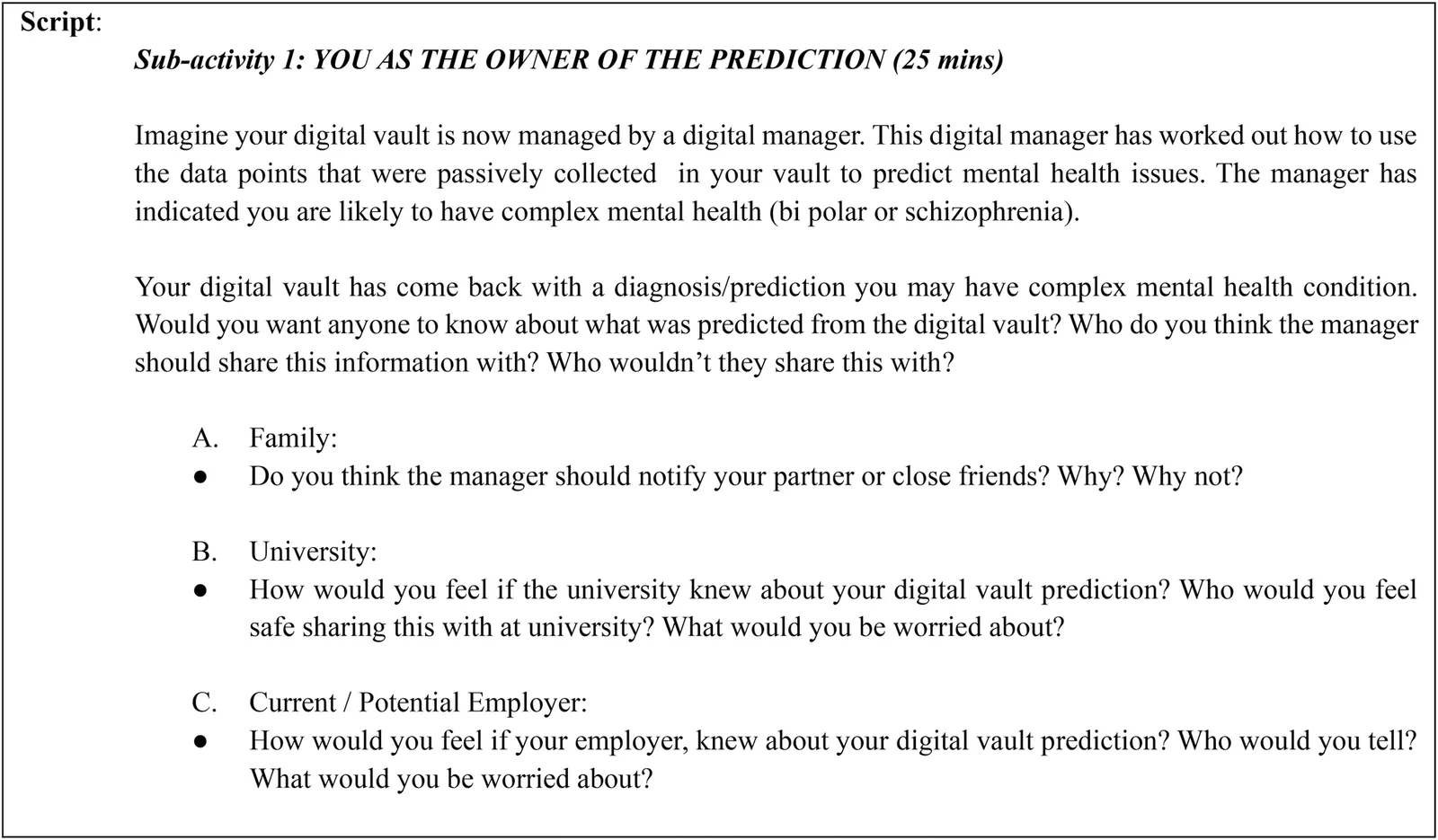
Research scenario nnnfrom activity 3.

**Table 1:** Participant Demographic

Participant Identifier	Age range	Cultural background	Languages spoken at home	Geographic location
P1	60–70	Indian	English, Tamil, Hindi	Metro
P2	50–60	Indian	English, Tamil	Metro
P3	50–60	Indian	Tamil	Metro
P4	60–70	German	German, English	Metro
P5	40–50	Italian	Italian, English	Metro
P6	50–60	Indian	Hindi, Punjabi, English	Metro
P7	60–70	Australian and German	English, German	Metro
P8	50–60	Australian	English	Regional
P9	50–60	Australian	English	Regional
P10	30–40	Australian	English	Regional
P11	30–40	Sri Lankan	Tamil, English	Regional
P12	50–60	Australian	English	Regional
P13	20–30	Australian	English	Regional
P14	40–50	Australian	English	Regional
P15	40–50	Australian	English	Regional
YP1	22	Turkish	Turkish	Metro
YP2	16	German and Indian	German, English	Metro
YP3	22	Malaysian	Cantonese, English	Metro
YP4	21	Indian	Hindi	Metro
YP5	23	Han Chinese	Mandarin Chinese	Metro
YP6	22	Australian-Chinese	Mandarin Chinese	Metro
YP7	18	Malaysian	English	Metro
YP8	24	South African	Zulu	Metro
YP9	24	Malaysian-Chinese	Cantonese, Mandarin, Bahasa Melayu	Metro
YP10	21	Indian or Hindu	English, Tamil	Metro
YP 11	18	Australian	English	Regional
YP12	17	Nigerian	Yoruba, English	Regional
YP13	15	Nigerian	Yoruba, English	Regional
YP14	16	Australian	English	Regional
YP15	15	Australian	English	Regional
YP16	17	Spanish	English, Spanish	Regional

### 3.2 Ethics and recruitment

Our study received ethical approval through Monash University. All participants were provided an explanatory statement and consent form prior to the session and their demographic information was collected once consent had been received. Participants were renumerated AUD $40 p/h for their time. In each workshop, we organised a co-facilitator who identified as part of the local community to reduce power imbalances between the researcher and participants and to foster safety and openness.

### 3.3 Contextual enquiry through user-centred design

We conducted 4 workshops: one with young people living in greater Melbourne (*n* = 10), one with parents of young people living in greater Melbourne (*n* = 7), one with young people living in regional Victoria (*n* = 6), and one with parents living in regional Victoria (*n* = 8) (Table 1). The participants were not related to one another nor were they receive acute mental health support at the time of the workshops. The participants were from diverse backgrounds including parents who identified as Australian (*n* = 8), Indian (*n* = 4), German (*n* = 1), Italian (*n* = 1) and Sri Lankan (*n* = 1), and young people who identified as Australian (*n* = 3), Chinese (*n* = 2), Malaysian (*n* = 2), Nigerian (*n* = 2), Spanish (*n* = 1), Turkish (*n* = 1), South African (*n* = 1), German/Indian (*n* = 1), Australian/Chinese (*n* = 1), Malaysian/Chinese (*n* = 1). Ther was a variety of languages spoken at home, including English (*n* = 23), Tamil (*n* = 4), Cantonese (*n* = 3), Mandarin (*n* = 3), Hindi (*n* = 3), Yorba (*n* = 2), German (*n* = 2), Zulu (*n* = 1), Bahasa Melayu (*n* = 1), Italian (*n* = 1), Punjabi (*n* = 1). A majority of participants were from Asia (*n* = 14/30, 46.7%), reflecting the demographic compostion of the Australian population (106). See Table 1 for all demographic details.

Specifically, we examined three research questions:

1. What are the barriers to mental health care service access for parents (in relation to accessing services for their child) and for young people living in different locations?
2. How do parents and young people value data about mental health, who would they share it with and how may it differ in between the generations?
3. What are parents and young people's preferences for engaging with DMH tools to support youth mental health and how can they be designed to suit their needs?

The workshops were designed to explore parents and young people's perceptions of the potential use of their data in the future. The sessions were guided by a semi-structured activity guide with an example of the activity guide seen in in Figure 1. These activities were designed with the intention of unpacking the participant's understanding of data security, access, sharing, custodianship or who owns the data, and potential data re-use. The multimodal approach including physical artefacts and drawing activities centred around a narrative continuum of an imagined digital data vault was designed with the intention to make abstract ideas more accessible and facilitate deeper reflection. This project was part of a larger body of work aiming to gather perspectives from under-represented populations. The same workshop format and methodology was conducted with each set of participants and is described in further detail in (15).

The workshop procedures were consistent across all four sessions, with minimal variation, as facilitators adhered closely to the provided activity guide. The first activity involved an icebreaker designed to elicit participants' conceptualisations of mental health and their experiences and perspectives regarding mental health services, thereby providing data relevant to RQ1. Using a whiteboard and pens, participants were invited to suggest aloud what they considered to represent (a) good mental health and (b) poor mental health. Although this activity was not designed to directly elicit barriers to accessing mental health services, participants' reflections and subsequent discussions provided opportunities for barriers and facilitators to emerge organically. Facilitators deliberately did not ask participants to identify barriers explicitly, as we sought to avoid framing the discussion in overly directive terms that might constrain responses or position participants as passive recipients of care. Instead, the activity privileged participants' own constructions of mental health and experiences of service use, allowing issues related to access and engagement to arise from participants' accounts. Accordingly, the findings relating to RQ1 should be understood as reflecting barriers that participants spontaneously raised or that could be inferred from their discussions, rather than as an exhaustive account of barriers to accessing mental health services.

Next, we provided participants with a speculative design probe (107) that we called a “digital mental health vault” (Figure 2, 3) to unpack abstract conceptions of digital mental health data to address RQ 2. The vault was a tin box that had 3 layers of security: (1) a locked safe using a metal box (high security), (2) an inner layer using a thick metal chain (medium security), and (3) an outer layer using a thin rope (low security). The choice to develop a physical artefact was designed to develop a shared reference point that facilitated discussion across diverse participants, reducing the reliance on technical or abstract language and instead anchoring dialogue in a metaphor that was accessible, interactive, and resonant. To this end, the vault functioned not only as a design probe, but also as a methodological device to elicit rich, more nuanced insights into the social and emotional dimensions of sensitive data in digital mental health contexts.

**Figure 2:**
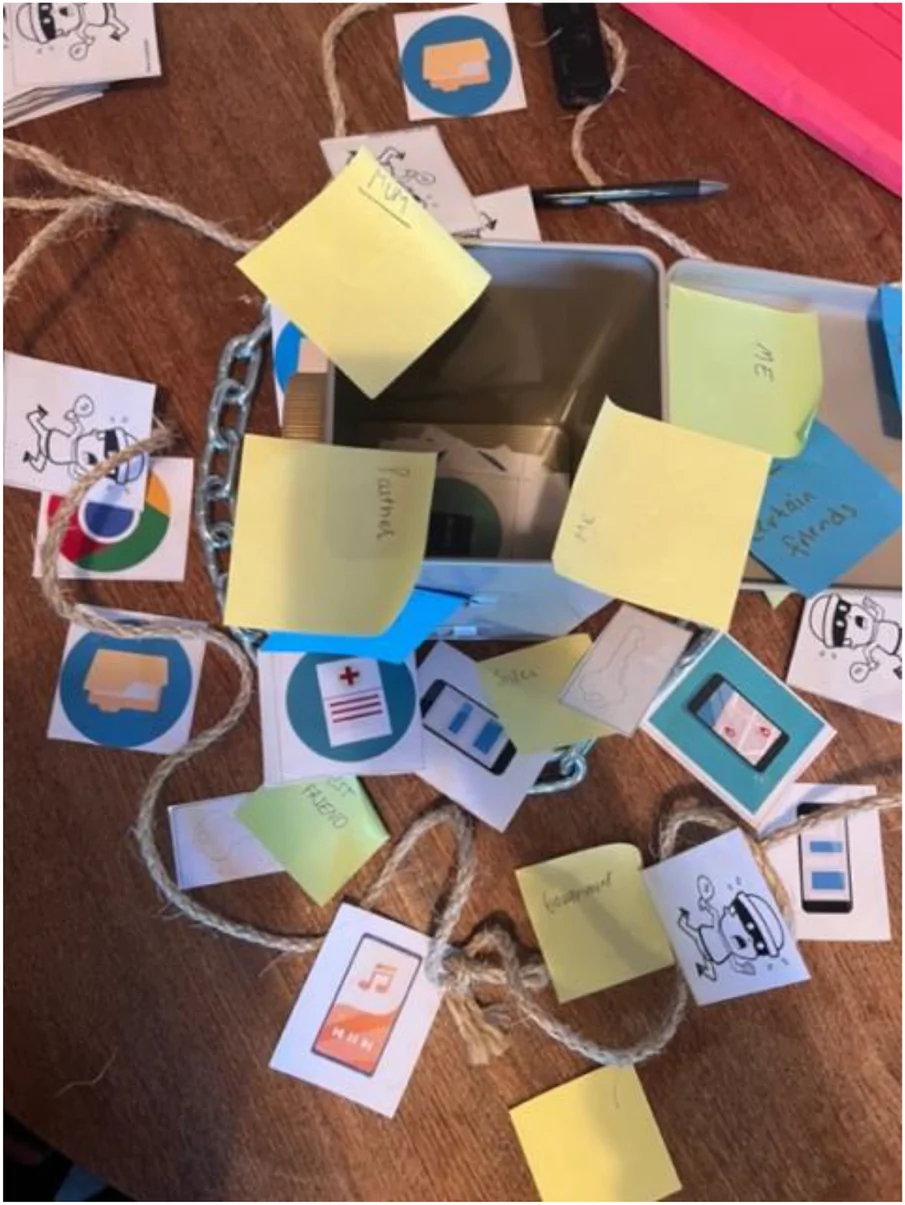
Image capturing the “digital vault” activity in workshop 1.

**Figure 3:**
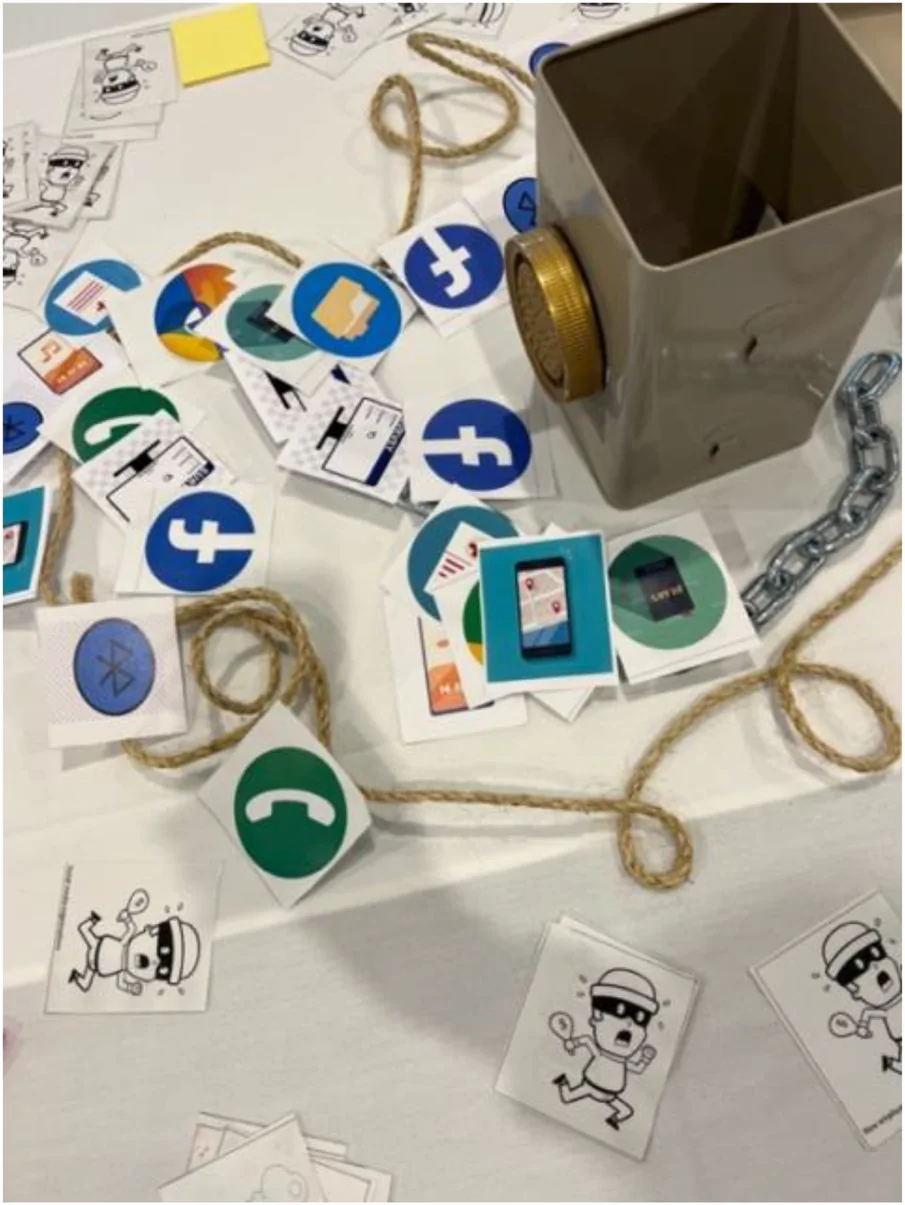
Image capturing the “digital vault” activity in work shop 4.

First we wanted to gauge how secure participants believed their mental health data might be by providing stimuli picture cards of potential mental health data points (e.g., a phone, survey test results, GPS data) and asked participants to place the stimuli pictures between the chains and rope that were placed on the outside of the mental health vault to signify the level of privacy (i.e., inside the metal box, inside the chain, and the outer thin rope layer). The most private was the inside of the metal box and the least private was the outer layer of the rope. Next, we provided participants blank paper keys and asked who the participants were willing to share their data with (i.e., peers, siblings, spouse). We then supplied participants with images of a burglar with a blank space below and asked them to write down who they thought might rob the vault. Participants then placed the newly labelled keys into the layer of the vault they felt the “data robber” might access. This activity helped participants to start considering who may access or “steal” their data. Building on this activity we introduced a “digital vault manager” who had access to the data contained in the digital vault and could use it to predict mental health issues. This activity was designed to get participants to consider their individual perspectives as well as how others may respond to having their personal data shared. We included schools and employers as potential data recipients to reflect the long-term implications of youth data sharing. Young people's engagement with digital tools in education settings may result in data being stored and potentially accessible for many years thereafter. Employers and schools represent realistic contexts in which personal data may later influence employment opportunities or decisions for enrolment and support. Considering these categories acknowledges that end users may be unaware of the inferences derivable from their data, which can reinforce biases, affect access to care, or shape perceptions within broader institutional systems (e.g., increased insurance, punitive institutional measures or lower employability) (108, 109). Finally, we explored how predictive information is communicated, understood, and applied in clinical practice by asking the participants to imagine the digital vault had been passively collecting their data for 20 years and had the capability to predict their mental health status. Through this discussion-based activity we explored how their prediction may influence trust and how the prediction could best be communicated and by whom. During this final activity, we probed participant's about how they would like the tool be designed in a way that would increase their trust to engage with it and whether they had any specific design recommendations or preferences.

### 3.4 Positionality statement

The authorship team for this project come from a variety of different backgrounds and life experiences. As authors, we acknowledge how our own cultural and gender-based beliefs and attitudes can influence both the study design and the way we analyse and interpret the data collected within this study. We took on this research as we personally believe that there is great importance in fostering equal opportunities to access mental health services for parents and young people and including them in inclusive designs of DMH technologies. The breakdown of the authorship team is as follows: 3 females with European, Anglo-Celtic and Irish heritage, 1 female with Southeast Asian heritage, and 2 males of Anglo-Australian and Filipino heritage. The authorship team consists of a clinical psychologist, two registered psychologists one working on DMH interventions for parents of children and young people, a neuroscientist and bioethicist working in DMH and responsible innovation, and two HCI and design practitioners. Three of the authors are parents and three work with young people, caregivers and families. The authors were committed to extending their own knowledge and understanding of diverse research practice to empathise and engage with parents and young people from different socio-cultural backgrounds in this work. This has informed our approach to the study design and data analysis. We feel that the diversity in our socio-cultural factors and individual experiences aided in our understanding, shaping the design methods for the workshops and enriching our data analysis.

### 3.5 Data analysis

All four workshops were audio recorded, transcribed and thematically analysed using NVivo 11 software. An inductive thematic analysis was conducted on each of the transcripts, without a pre-existing coding framework, to explore the interpretations of common themes and subthemes (110). First, four members of the research team (IB, RM, AC, JS) familiarised themselves with the data by examining the transcripts and creating points of interest. The points of interest were then considered, and initial codes were generated by IB to organise the data (110). A final round of cross coding was completed to refine themes (IB, AC, RM) (110). Codes were reviewed for redundancies and overlap before the final themes and subthemes were developed and agreed upon in consultation with all authors. The coded data from all four workshops were combined for analysis with 3 overarching themes developed. In each workshop an activity schedule was used to scaffold free-flowing discussion among participants. Differences across participants' perspectives were examined through the coding process and used to identify and refine subthemes. Group discussions encouraged participants to share distinct cultural perspectives and lived experiences, generating diverse and detailed accounts throughout the sessions.

## 4 Results

We identified 3 overarching themes regarding parents and young people's perspectives on DMH tools: (1) Attitudes towards mental health and how it is discussed, (2) Trust in DMH tools, (3) Design preferences of parents and young people.

### 4.1 Attitudes towards mental health and how it is discussed

#### 4.1.1 Mental health disclosure and the family unit

While prior research has well established the importance of family communication and mental health literacy in shaping help-seeking behaviours, our findings reaffirm these dynamics while highlighting a more nuanced relational dimension. Specifically, relationships grounded in openness and psychological safety were more amenable to mental health disclosures, whereas parental insecurity, shame, and secrecy created barriers to open discussions within the family unit. Young people frequently perceived their parents as lacking the knowledge or emotional readiness to engage with mental health issues or were not qualified to help: “*My parents didn’t study to become parents. So I feel like they’re less qualified on certain topics. They’re not qualified to help me [.] to help people for mental health.”* (YP11, 18, regional). Additionally there was a sense of judgement when they did eventually share or disclose challenges: “*Somehow they managed to be judgmental and over reactionary and downplay your mental health at the same time*.” (YP5, 23, metro). Instead young people often redirected their disclosures toward peers or professionals: *“But going to a friend, or a therapist, or a doctor can sometimes be really good because you can get the right advice. Sometimes going to a parent is just, some things you don't want to go to a parent about.”* (YP11, 18, regional).

How mental health was discussed with others appeared to be an important factor and was likely informed by the intersectionality of the participant backgrounds. Parents described feelings of guilt and denial, with some reporting that they would avoid telling the child's siblings, extended family or professionals about their child's mental health when difficulties first present. Participants in both the regional and metropolitan parent groups described how their children's preferences for support and whom they rely on change as they move through different developmental stages. The relationship between the co-parents also played an important role where parents “*become competitive with one another*” (P9, 50–60, regional). For the parents in the metropolitan group, stoicism, stigma within the community, and concerns that “*mental health [was] less measurable compared to physical health problems*” (P7, 60–70, metro) were barriers to help seeking. In contrast, most of the participants in the regional parents' group had lived experience of complex mental health diagnoses and neurodiversity and reported that they were raising children with similar lived experiences. The regional group reported that they needed more resources and information on raising children with mental health challenges, particularly for raising neurodiverse children. However, for some, online sources contained misinformation: “*In the digital world, we’ve created a problem where there's too much conflicting information. So where you’re getting your information from is just as important as the information itself.”* (P9, 50–60, regional). Regional parents in particular felt blamed for their child's struggles with little optimism from local specialists or services. One parent described her experience: “*A lot of specialists, get stuck on deficits and don't start talking about the positive aspects.”* (P10, 30–40, regional).

In summary, participants described how open, trusting family relationships enable mental health disclosures. In contrast, parental shame around mental health and fear of judgement impeded conversations, prompting young people to seek support outside of the family. Parents' own experiences, challenges finding reliable information and resources online, differences in mental health literacy, and socio-cultural factors (i.e., stigma) shaped how mental health was perceived and addressed across both metropolitan and regional contexts.

#### 4.1.2 Mental health disclosure and societal influences

Parents in both regional and metropolitan groups reported lived experience of personal discrimination based on their gender, status as a parent, as someone with mental illness, or based on their culture. Regional parents reported feeling dismissed by their community and services based on their own or their child's mental health, “*I'm a mother and I've got mental health issues. I've known those mental health issues have [been] used against me”* (P10, 30–40, regional), including via child protective services, employers, real estate agencies and family members. Some members of the regional parents' group had used the National Disability Insurance Service [national social services supporting people with disabilities] and discussed how they felt like they were treated “*like second class citizens with a disability”* (P13, 20–30, regional). A preference to keep health and mental health care separate was also identified by parents, with two parents in the regional group discussing concerns that their mental health diagnosis could impact how their physical health was understood and treated. One parent made sure her general physician did not know or contact her mental health practitioner:

“So if I cut my finger, I'll see that doctor. I don't want that doctor knowing about the other stuff. That's none of his business. That's why I've got two doctors. One that I can be crazy with and myself, and one for boo boos. So, I like to keep my records sealed. So when you said that thing about linking it all, I'm like, I don't think so. That's important because I think one doctor has the right to know about booboos, and the mental health doctor is a mental health doctor. And if all doctors can have it on there, then they're all going to think I'm crazy.” (P11, 30–40, regional)

This preference expressed by a number of participants to keep their physical and mental health separate could have implications for DMH tool design. Importance was placed on spiritual influence across all four groups, in particular how culture and faith informed the parent-child relationship, providing hope, helping to make sense of things, and referring to religion to help with moral issues. One young person explained:

“I've found at church and stuff, the Bible [.] has a lot of talk about, on a lot of moral issues and ethical issues, and as a Christian, because I choose to be a Christian, I choose to follow those sort of things and to follow those mental health the things it recommends you to do and stuff.” (YP16, 17, regional).

Another parent identified religious music as helping with her parenting:

“My faith is very important to me. Because parenting is such a tricky and challenging task, despite the fact there are more rewards. One needs some sanity to keep oneself patient. And I found music was the thing, and Christian inspirational players were what worked for me.” (P12, 50–60, regional).

These findings illustrate how spirituality and religion can operate as socio-cultural resources that underpin community and mental health resilience. Across both settings, faith-based practices and moral frameworks were described as offering mechanisms for coping, hope and meaning making. Despite the perceived stigma and discrimination, participants across both parents and young people groups agreed that conversations and awareness around mental health was becoming more normalised. Regional parents described living in close knit communities, with two participants who had neurodiverse children explaining that their children educate their small rural community on Autism Spectrum Disorder (ASD):

“We live in a rural community out in [redacted]. We go to the little supermarket which is in the next little town along. And my son educates people about autism quite loudly on a regular basis. And, and I've found that, we still get the looks, all of that still happens. You know, but it's just our attitude to it. Yeah, it's just educating people about things that happen to us.” (P12, 50–60, regional)

In summary, participants across both metropolitan and regional groups reported intersecting experiences of discrimination related to mental illness, parenting, and culture, prompting some to separate their mental and physical health to avoid prejudice. Despite these challenges, cultural and spiritual frameworks provided coping resources, along with increasing efforts to normalise and create awareness of mental health and wellbeing in regional communities. For the design of DMH tools, this suggests the importance of recognising and where appropriate, considering how spiritual values and language can support and resonate with user's cultural and spiritual world.

### 4.2 Schools as a friend or foe

School systems and their level of support and surveillance was discussed across all four groups. Some young people felt that school was supportive, mentioning the usefulness of wellbeing check ins such as “*Wellbeing Wednesdays”*(YP12, 16, regional). They described feeling close to their teachers and school counsellors and relying on them as confidants: “*Teachers are a lot closer than most other people in your life. Yeah, you spend a lot more time with them [.] They, like, have the reason to be able to check in and just ask, are you feeling good?” (YP16, 17, regional)*. In contrast, other young people felt uncomfortable about sharing their mental health with teachers, with one elaborating:

“I wouldn’t really go to a teacher anyways, I had a problem I would go to friends or a counsellor. If my teacher asked how my mental health was going, I. would feel a bit uncomfortable sharing it with him, because he would probably bring it up in a random humanities class and be like ‘oh that's right this reminds me of your mental health”. (YP14, 16, regional)

Participants discussed how teachers did not have the same legal and ethical obligations as psychologists, and young people reflected that they would avoid disclosing serious mental health concerns with teachers, afraid that their duty of care guidelines could mean they break confidentiality and alert a young person's parent or caregiver. On the theme of confidentiality and privacy, some young people highlighted the use of school laptops and reflected on whether the school has the right to surveil students (e.g., to monitor homework). Some participants believed that they did whilst others felt it could be used against them: “*Schools might use it to see why you're not doing your schoolwork, or why your schoolwork is not up to scratch. But if the school's doing it, that's a bit sketchy.”* (YP13, 15, regional). Parents, however, reported not feeling listened to by teachers: “*The teachers won't listen to what we say. They don't do anything, you know, we've got the child for, they've got it for six hours a day, but we've got the child for the rest of the time. But the teachers won't listen to what we say.” (P14, 40–50, regional*). An example was given of teachers not letting neurodiverse child use appropriate soothing techniques in the classroom: “*We're having an issue at the moment in primary school… Our son's not allowed to use his chair to help him regulate because it distracts the other children.”* (P13, 20–30, regional). Parents in the regional group agreed that younger teachers were viewed as more accommodating for neurodiverse children whilst they faced more challenges with older teachers. One parent explained: “*We've found the older teachers are blocking them from putting that knowledge [about neuro-affirming teaching styles] into practice. The younger teacher's know about it, they know how to flag it. The older teachers are like ‘we don't to do that'.”* (P12).

### 4.3 Trust in digital mental health tools

The study found that both parents and young people preferred to keep their identities private, were aware of their data being used by third parties and had contrary attitudes toward passive data collection by DMH tools (e.g., wearables, smartphones) in a clinical setting.

### 4.4 Data privacy and separate personas

Parents' groups reported an eagerness to keep their medical records and information relating to services private. Fears of bank scams, data breaches, insurance premium increases, discrimination by social services, and identify theft were commonly raised. Members of the parental groups stated they believed medical data should be kept private, with some stating they used two phones to avoid scam phone calls. Parents were mindful that their data could be used by third parties, elaborating “*The data universe knows my parenting status. They know I'm a single parent, they know I have a child with a disability*” (P9, 50–60, regional). Two parents reported being victims of previous online scams or data breaches whilst others expressed concerns for how healthcare data may be exploited or shared with third parties to reveal personal information.

“So if I think the technology's okay, I really trust it. But at the same time, I've got children and I've been hacked. So it depends. I do trust Centrelink [government department that delivers income support and services to Australians] and I do trust My Health Record [a national government health record for each citizen]. But I don't trust, you know, and having children on there and keeping them safe. So it's a tricky one in terms of the internet.” (P13, 20–30, regional)

For parents there was more of a focus on the impact of how their data could impact their family compared to the young people who were concerned about online and commercial third party interactions. Young people were worried about their personal information being shared, stolen or leaked, describing feeling “*taken advantage*” (YP2, 16, metro) by commercial third parties, identifying tech companies as “*closed ecosystems*” (YP11, 18, regional) with one young person stating: “*Whoever's in control of all this information, all this information has control over your life basically*.” (YP16, 17, regional).

Another expanded: “*They learn more information about me in order to affect and influence other people's behaviors as well. And I personally find this really wrong. I know we cannot escape this at this point, but I don't like participating in this culture at all. And I personally don't like the idea of looking up a music that I really like and it being targeted at me for other purposes, like marketing purposes or analytical purposes.”* (YP1, 22, metro).

To manage this sense of surveillance, each group reported separating their identities or creating online personas to avoid government surveillance or other actors (i.e., tech companies, police force) from having total control of their data. One parent explained she uses two phones and fake emails to increase her privacy: “*I'd purposely make a fake email to see what they're wanting to talk about. I've got a separate phone, which I know because I'm so paranoid about people calling me, I'll actually text that number asking what it is all about.”* (P13, 20–30, regional). Another stated she avoided using her smartphone or Google Maps: “*With google Maps and stuff, I don't allow it to know where I'm going because I don't want anyone to know where I am. You can go where you like, just keep your phone at home, no one knows you're out. So there's different ways to hide yourself from the world.”* (P5, 40–50, metro). Using online personas was a mode of regulating their online activity and behaviour, with participants stating the need for a persona had increased over the years since engaging with the digital world. One participant noted the use of personas and performativity was increasing: “*I think just now more than ever, the online landscape now compared to 10 years ago, it feels like all the personas are performing. Everyone online is performing for an audience.”* (YP5, 23, metro).

Another young person elaborated and stated in relation to the effectiveness of DMH tools when gleaning accurate information based on online data: “*I don't believe an app can get enough information to accurately tell someone they've got complex mental health. You know, that person could be portraying a persona online, they could be the happiest person in the world, or they might be very depressed but be a happy persona. It's easy to portray a persona online that doesn't reflect the person”* (YP13, 15, regional). Similarly, the concept of difference between someone online and offline was considered, with one young person reflecting that “*The online space has its own demographic, like you're performing. And in addition to that, everybody has an audience that know that they will side with them. So the online atmosphere is quite different to the real world. Somone might who might think they will be judged in the real world, can express themselves freely on the internet. But at the same time, somewhere else online there's going to be pressure for someone to be silent.”* (YP10, 21, metro). The observation of an online persona was relevant for both youth groups, with a consensus that online personas were protective in the face of digital harms, it was a mode of freedom of expression, and to maintain anonymity.

On one hand young people enjoyed the freedom of the internet, “*The online anonymous aspect made it a safe place for me to voice my mental health concerns about myself”* (YP6, 22, metro). On the other, they were concerned about how the internet could be an echo chamber, that algorithms could inform political views, and that the online atmosphere was different to the real world. One young person explained:

“A lot of the videos that I click on, it's always like echoing what I'm thinking as well. Kind of like an echo chamber, in the comments. Then it's always reinforcing exactly what I think, it's never seeming to like to contradict what I see. You know some people don't believe that global warming exists because they don't see it anywhere at all” (YP2, 16, metro).

### 4.5 Perceived limits of AI and the value of human connect

Whilst the activity guide did not specifically probe participants on artificial intelligence (AI), participants conceptualised digital mental health tools to involve a level of AI input, with members in each group considering different computing systems to be required for DMH tools to function. There were shared concerns that mental health was too complex for AI or that AI could misdiagnose, and that an app may not detect the nuance of daily changes in mood. One participant expanded:

“I don't think AI can accurately tell you have A, B and C. Because there could be a lot of things mimicking it as well. A lot of things have very similar symptoms. And they could completely misdiagnosis and then go, Oh shoot, you're actually this. You're not, or you're both. You have symptoms that match, but we didn't see the rest.” (YP15, 15, regional)

The importance of face-to-face interaction for therapy and the delivery of a diagnosis was explored, with young people agreeing that human connection was needed in therapy, with one young person stating that “*apps can only do so much. Like personally, I'd say you get something more out of a human interaction”* (YP10, 21, metro). Similarly, another young person explained they would prefer to have a diagnosis or future risk notifications delivered in person:

“It doesn't feel like you're sharing time with a person, because we're social creatures, we want time with other people. So yeah, there's like that knowing sense of, ‘Oh, this is all fake. Why am I doing this?’ So yeah, so I feel like in 20 years' time, it'd probably be more important to meet them [clinician] face to face rather than through monitors or screens.”(YP9)

Young people questioned whether DMH tools would be able to capture the complexity of the human experience:

“I don't feel like you can have auto generated apps and stuff, because for mental health, everything is a spectrum. Autism, ADHD, bipolar, schizophrenia, um, anything. PTSD, it's all a spectrum. And people fit on that spectrum. I feel like you can't have an app that says, ‘You are at a 2 on this spectrum'. Because some days you might be at a 2. But some days, you might be at a 10. Some days you might be at a negative 1. You never know.” (YP11, 18, regional).

Another young person echoed this and reiterated the performative aspect of online personas:

“I don't believe an app can get enough information to accurately tell someone, ‘yep you're dealing with mental health, yep, you're gonna deal with mental health'. I think mental health is such a complex thing. You can't just base it off of someone's music type, or how they talk, or how they are online, because there's a lot more behind that screen that you can't see. You know, that person could be, portraying a persona online, they'd be the most happiest person in the world, or they might be very depressed but be a happy person. Like, it's easy to portray a persona online and I don't think an app is going to be able to tell me, and today you're going to be depressed. Unless it can physically read my brain.” (YP10, 21, metro)

Young people had varying attitudes toward passive tracking by remote sensing tools (i.e., smartphones or wearables), with some arguing that it could be useful to manage mental health episodes or addictive behaviour patterns, as one young person explained*: “I know we've been talking a lot about like as a diagnosis tool, but, or like prognosis tool, but maybe it could also be as like used as a tracking tool. And like how we were saying for like a bipolar disorder if you know you have a mental health issue then you could use it to track it and manage that.” (YP2*).

Others suggested it may increase paranoia*: “I feel freaked out if an app told me, oh, you're at risk of committing suicide because I'm like, okay, what else do you know about me?”* (YP8, 24, metro). Some argued those with complex mental health are less likely to be using an app: “*I think maybe for people who are, who are already suffering from mental health issues, I think it might be a little bit more difficult to get them to use the software.” (YP2, 16, metro).* Furthermore an app's functionality was scrutinised by some young people, arguing that unlike their parents, an app cannot force the user to see a psychologist, or that an app cannot predict daily interactions that may upset the user. This was explained by one young person:

“You don't know what the future holds. An app can't exactly go, ‘oh yes and at 4. 59pm today you're going to get a text from your best friend saying, Sorry, I'm not friends with you anymore'. And I can't predict that, and that's going to affect your mental health. That's going to affect your emotions entirely.” (YP7, 18, metro)

Other young people were concerned that phone patterns may be inaccurate and lead to inappropriate assessments based on the passive data collection:

“What if it's economical issues? And not necessarily that I'm depressed. I'm having less interaction with my friends because of economical issues. Or what if I can't afford my Optus plan? And I'm no longer on my phone. You know what I mean? What if I can't afford my Wi Fi?” (YP8, 24, metro).

### 4.6 Design preferences of parents and young people

After discussing the barriers to engaging with mental health services, levels of trust in DMH tools and what they may do, participants reflected on their own preferences for engaging with a DMH tool and how it may bridge the gap with their current mental health care. They explored the features they would like in a DMH tool and whom they would like to share or use the tool with, and shared their reflections on usability.

### 4.7 Young people want a tool that is centralised with their caregivers and providers

When exploring how DMH tools may be developed and designed to accommodate young people, a centralised app was workshopped that included guardians, family members, teachers, healthcare professionals and other members of a care team that received updates on the young person's mental health data with universal access for the chosen contacts. They imagined an app that could triage a user based on urgency, offering resources and support for both the user and the nominated contacts and that when the nominated contacts joined the app, they accepted some responsibility for the young person's wellbeing. Some young people preferred the idea of the app providing broad support, offering recommendations and support rather than a diagnosis*: “The limiting factor is everything is so broad and people are a spectrum and its gonna be useful for some people and its gonna be not useful for some people (YP5, 23, metro)*. Some suggested it would act ‘*like an icebreaker’”* (YP16, 17, regional) to broach conversations about mental health with their family. Others highlighted how it could increase access to mental health support: “*Making a difference in the equity by allowing people who do have that access to get further ahead with their mental health.” (YP2, 16, metro).*

Another participant described a centralised app as a 'share house' whereby parents, carers, teachers, mental health clinicians were linked into a centralised app:

“Like a share house [living accommodation shared with friends] let's say to download the app, it would relate [be the equivalent] to this person living in the house [.] People work like a team. Maybe those who aren't paying the bills or something, they may get kicked out of the house [.] And likewise with the people that are just using the app to spy on you, not helping you at all, then kick them out as time goes on.” (YP16, 17, regional)

Young people's strong desire for privacy and separate identities was at odds with their interest in a centralised, integrated support network, which inherently requires disclosure. This tension underscores the challenge of balancing confidentiality with the benefits of connected digital mental health support and may reflect limited awareness of the data needed for effective tool design.

Despite the concerns and challenges about whether digital tools could be accurate, evidence based, and as supportive as their human counterparts, overall there was an openness to trialling DMH tools provided that it occurs alongside face to face care. “*I just want the results in person. And then after that you might be interested in doing things that are offered digitally. You want someone to actually show, hey, you're not alone in this. You've got a team, and we're going to help you.”* (YP11, 18, regional)

Parents shared a similar view and were open to using DMH tools to share the results or recommendations provided by the tools with their healthcare professional, so that they could trust the results provided by the tools: “*I think it's for my GP. I'm to discuss it with my GP first. Then, with that information, keep researching and take it one step at a time to seek help, I guess. To prevent it from getting worse. Early intervention. […] Yeah, because I think with that information I can make adjustments*.” (P5, 40–50, metro).

### 4.8 Parents want a tool that is personalised, targeted and includes resources

DMH tools were appealing to parents, with some benefits including prevention, individual and tailored support options, access to services instead of having to drive long distances, reduced wait-times and not needing to pay expensive fees for specialists. Before parents would consider engaging with a DMH tool, the requirements for a high level of trust and credibility in the technology was voiced across both groups, “*I think the biggest thing for me with technology is it's got to do what it says it does*” (P9, 50–60, regional).

The idea that DMH tools may help prevent poor mental health was discussed, with one parent expanding: “*I mean, look, in an effect, prevention is better than cure. If you could be warned about something, So, literally, you put that extra insulation around the kid.” (P6, 50–60, metro)*

Despite highlighting the importance of separating their identities and medical information, parents agreed that a streamlined DMH tool that included chosen contacts, could help healthcare practitioners and teachers to listen to parents and understand their children's needs, despite feeling invasive: “*Yes, it might be a little bit invasive, but you have less worry or stress to try and figure out what your kid needs*.” (P10, 30–40, regional). They suggested having an option to revoke permission to access medical records or mental health data, but that having centralised records and data collection would be useful for complex issues. Parents stated a DMH tool would be useful if it was evidence based, the data remained anonymous, and forewarned parents about how it would notify them of changes in their child's mental health. For example: “*As part of logging into the app, it says in the future, ‘We'll be in touch if something in your records indicates something’”* (P9, 50–60, regional).

As a result of the discrimination and stigma parents had experienced, they reported having to learn how to advocate for themselves when interacting with professionals and services. Parents were eager for a DMH tool to include resources that helped to teach self-advocacy and connects parents with others:

“If you, if your tool could connect us to people. You can teach them how to self advocate. That should probably be your first option in the tool, is to, [teach the user] how to self advocate. I think you need to guide them first and then you can give them information.” (P13, 20–30, regional).

While prior research has well established the importance of family communication and mental health literacy in shaping help-seeking behaviours, our findings reaffirm these dynamics while highlighting a more nuanced relational dimension. Specifically, relationships grounded in openness and psychological safety were more amenable to mental health disclosures, whereas parental insecurity, shame, and secrecy created barriers to open discussions within the family unit. Young people frequently perceived their parents as lacking the knowledge or emotional readiness to engage with mental health issues or were not qualified to help: “*My parents didn't study to become parents. So I feel like they're less qualified on certain topics. They're not qualified to help me […] to help people for mental health.”* (YP11, 18, regional). Additionally there was a sense of judgement when they did eventually share or disclose challenges: “*Somehow they managed to be judgmental and over reactionary and downplay your mental health at the same time*.” (YP5, 23, metro). Instead young people often redirected their disclosures toward peers or professionals: *“But going to a friend, or a therapist, or a doctor can sometimes be really good because you can get the right advice. Sometimes going to a parent is just, some things you don't want to go to a parent about.”* (YP11, 18, regional).

How mental health was discussed with others appeared to be an important factor and was likely informed by the intersectionality of the participant backgrounds. Parents described feelings of guilt and denial, with some reporting that they would avoid telling the child's siblings, extended family or professionals about their child's mental health when difficulties first present. Participants in both the regional and metropolitan parent groups described how their children's preferences for support and whom they rely on change as they move through different developmental stages. The relationship between the co-parents also played an important role where parents “*become competitive with one another*” (P9, 50–60, regional). For the parents in the metropolitan group, stoicism, stigma within the community, and concerns that “*mental health [was] less measurable compared to physical health problems*” (P7, 60–70, metro) were barriers to help seeking. In contrast, most of the participants in the regional parents' group had lived experience of complex mental health diagnoses and neurodiversity and reported that they were raising children with similar lived experiences. The regional group reported that they needed more resources and information on raising children with mental health challenges, particularly for raising neurodiverse children. However, for some, online sources contained misinformation: “*In the digital world, we've created a problem where there's too much conflicting information. So where you're getting your information from is just as important as the information itself.”* (P9, 50–60, regional). Regional parents in particular felt blamed for their child's struggles with little optimism from local specialists or services. One parent described her experience: “*A lot of specialists, get stuck on deficits and don't start talking about the positive aspects.”* (P10, 30–40, regional).

In summary, participants described how open, trusting family relationships enable mental health disclosures. In contrast, parental shame around mental health and fear of judgement impeded conversations, prompting young people to seek support outside of the family. Parents' own experiences, challenges finding reliable information and resources online, differences in mental health literacy, and socio-cultural factors (i.e., stigma) shaped how mental health was perceived and addressed across both metropolitan and regional contexts.

While both parents and young people expressed a desire for both privacy and an integrated support network or streamlined care, these preferences need not be strictly contradictory. Instead these results demonstrate an opportunity for personalised approaches, such as selective sharing as illustrated in the young people's ideas on an integrated support network, which could allow individuals to maintain confidentiality whilst still benefiting from centralised resources. By allowing young people to selectively nominate guardians teachers, and health care professionals to a DMH app, it could enable controlled disclosure while providing a connected support network. Features such as urgency based triaging and targeted resource sharing could allow young people to retain agency over their data, while nominated contacts can meaningfully engage in the user's care, creating a balance between confidentiality and integrated support.

## 5 Discussion

This study sought to understand the barriers to mental health service provision for parents and young people, especially those underserved by current mental health models due to their socio-cultural backgrounds, and how they value DMH data and who might they share it with. We also examined how future DMH tools could be designed to suit their needs of young people and their parents. Based on these findings we identified five key considerations for effective DMH design and innovation.

### 5.1 Social identities inform help seeking

Whilst informal sources for help seeking may be the solution for young people who feel that their parents do not understand their mental health challenges, the parent-child relationship and parental influence remains critical for help-seeking behaviours (111, 112). Many young people in this study described their parents as having limited mental health literacy leaving them feeling misunderstood, or that their parents were not trained to provide mental health support. This highlights a gap that DMH tools could address, providing the necessary psycho-education on mental health to reduce parental stigma, increase awareness, and provide supportive resources for parents and caregivers who are supporting a young person's mental health (83, 113–116). Such tools could act as a platform to share parent-child challenges and offer support and resources to help parents foster a closer relationship with their young person. Examples of this in practice include online parenting programs like Partners in Parenting, which were co-designed with parents, young people and professionals for early intervention and prevention (68, 83, 114, 117), that empower parents with parenting strategies and confidence to support their young person's mental health (118–120).

Parents from regional areas may find DMH tools especially beneficial due to a desire for more information about mental health and neurodiversity, but limited availability of resources for them. However, for these DMH tools to work, young people need to be open with parents about their experiences. This poses a challenge as the young people in this study expressed preferences for not sharing or disclosing to parents, which could then have impacts on the future use of digital technologies and surveillance.

As young people continue their journey from adolescence into adulthood, parents should coach rather than surveil their children, providing e-safety education and building trust and autonomy in the young person to use the Internet safely (121). However as parental surveillance begins to be promoted to protect children's safety online (122), parents can be placed in a complicated position of safely monitoring their child's behaviour whilst negotiating when to intervene in their child's online activity and break trust (123). Meaningful and nuanced dialogue between parent and child can help negotiate their online presence, privacy and surveillance (124, 125).

Given young people's concerns about their parent's ability to understand and support their mental health challenges, peer groups may provide an additional resource. Previous literature has explored the role that peers play in supporting other young people, with peers being highly influential during childhood and adolescence (125), with many young people displaying a preference for discussing their mental health with friends (126). Peer-to-peer support can be effective for improving an array of mental health challenges (127, 128); however, the quality of online support and information can be inconsistent, with variations in delivery and efficacy (129). Studies have found that young people prefer to seek help from informal sources such as the Internet (130, 131), with those more at risk of suicidality or self-harm being more likely to access the Internet than reach out to someone (132). One example of this is the Moderated Online Social Therapy (MOST) digital platform that offers an integrated solution for young people including peer workers and a social network of peers (133, 134).

The parents in this study reported feeling dismissed by healthcare professionals, stigmatised by their local communities for having mental health challenges, and isolated for having children with disabilities or mental health challenges. This impacted their sense of self, their social identity, and the connectedness to their community. Their roles as either parents or young people, within their community, and the type of support they offered and received, was informed by their unique sense of identity. For example, the social identity of a single parent from a regional town who had poor experiences engaging with local and government services, including feeling dismissed by their child's teachers, informed her attitude toward help seeking. The parent participants were open to DMH tools despite stating that it would feel “*invasive*”. These collective feelings of “*invasion”*, “*denial”*, “*guilt”* that the parents expressed align with their collective experiences of discrimination and social exclusion. The parents identified as being from intersecting communities and their identities were expressed through religion, spirituality, socioeconomic status, and geography. Social identities provide individuals with a sense of purpose and belonging, thereby enhancing resilience against psychological distress, which is particularly significant for members of diverse communities who face discrimination or social exclusion (135). A person's sense of self is derived from their membership in one or more social groups (136). This is pertinent for young people who seek social inclusion and approval as they mature through adolescence (137). Cultural and community identity and connectedness are important for young people, who are still developing their sense of self, with research finding cultural identity can be a protective factor against suicide risk and depression (19). Furthermore, increased social identification (i.e., ethnicity, sexual identity, identification with neighbourhood) is associated with reduced symptoms of depression and anxiety (135). Intersecting social identities is important to consider for mental health service provision for young people who may be feeling ostracised from care based on their social identity and social positionality. For example, iBobbly, is a suicide prevention app designed for First Nations Australians that employs an Indigenous social and emotional wellbeing (SEWB) framework and incorporates traditional localised language (138).

Community stigma toward mental health meant that parents from this study were unable to talk about complex mental health with friends and family or were “*treated like second class citizens with a disability”* by government services. This discriminatory experience is reflective of Australia more broadly. The Australian Human Rights Commission 2022–23 report found that 46% of complaints they received were about disability discrimination (139). Disability-based discrimination has a strong association to psychological distress (140). There are risks with DMH tools further discriminating via the datafication and quantification of the human experience of everyday life (80), where the end user may be undermined and no longer considered the “reliable narrator” (141) of their own mental health experience. This is concerning when an automated decision system's functionality can be built upon inferring whether a person has an impairment such as a mental health condition, cognitive impairment or disability (142). How these systems are built to predict such outcomes can be informed by biases, discriminatory assumptions and stereotypes that could further ostracize end users (143–145). Furthermore, supplementing DMH tools as a technological solution for service provision can risk perpetuating the assumption that individuals are responsible for their own health outcomes, overlooking the structural and social causes of poor health (146).

Using social identities as a framework to help identify barriers to the uptake of DMH tool could help developers better understand how to design for parents and young people whose mental health perceptions are informed by their socio-cultural experiences such as discrimination and social exclusion.

### 5.2 DMH in the school setting

Schools can be the first port of access for young people struggling with mental health challenges. Both parents and young people in this study recognised the significance of how much time a young person spends at school, and the important role teachers play in supporting them. For most young people almost half of their waking time each day is spent with peers and teachers (147). Teachers regularly provide a form of primary care to children and young people and are often the first point of contact for young people with and without a mental health disorder (148). However, teachers have identified that they lack capacity, skills, and resources to effectively support students with mental health difficulties (149) and identified more training in early detection and intervention is needed (150). Furthermore, the teacher-student relationship has been found to predict general health including mental health into adulthood (151). Given the time spent and the powerful influence a teacher can have on a young person's life, it is important to consider how DMH tools could be used effectively within this setting. DMH tools could deliver support and increase service capacity (152); however, the young people in this study had contrasting attitudes toward classroom surveillance and the level of teacher involvement when it came to supporting their mental health. The inclusion of additional members of a young person's care team such as school counsellor, clinician or teacher could be complementary to an integrated support system. To reduce the perceived sense of school surveillance, teachers could be educated on the impact of classroom surveillance as well as increased institutional transparency, which could reduce the risk of young people self-restricting or self-censoring their behaviour to avoid feeling monitored or reducing freedom of expression (153, 154). When considering the utilisation of DMH tools, school counsellors identified benefits such as identifying at-risk students and providing psycho-education but have noted possible challenges in relation to gaining parental consent (155).

Some young people relied on their teachers as confidants whilst others were aware that their duty of care and responsibility was different to that of a psychologist. Young people were cautious toward the acceptability of school allocated laptops being used to monitor student activities. Knowledge that a user is being surveilled can cause psychological distress and lowered perceptions of safety (156, 157). Given the school setting is designed to discipline and assess, surveillance systems risk being designed or positioned to unfairly punish students (153, 158). Consideration for how the school setting could be involved in the design of DMH tools is important, particularly to improve the relational dynamics between parents and teachers. Lastly it is important to provide teachers with more mental health literacy and training so they can support young people as they progress through the education system.

### 5.3 Data governance and transparency

DMH tools that obtain, store or share medical and mental health data, pose risks for how the data could be used and shared without permission in the future. The participants were cognisant of the myriad uses of data and the powerful actors that store and share it. Researchers and designers have a duty of care to consider how the sensitive data that is produced from DMH tools is collected, safely stored, whether it is shared, and compliance with data security regulations (e.g., National Safety and Quality Digital Mental Health Standards in Australia (159) or the Medicines and Healthcare products Regulatory Agency in the United Kingdom (160)). The results illustrated a community level awareness of the potentiality of data dual use. Parents and young people spoke about their previous experiences of data misuse and their current actions to avoid future misuses, such as the creation of online personas. However, these actions taken to protect personal data could make it difficult to find a balance between gathering enough personal information to accurately use a DMH tool, whilst allowing an end user to maintain their privacy and preferred anonymity. We suggest that simple explanations are included when downloading or engaging with DMH tools, to explain where the user's data may be shared or stored to help users feel safe to share personal information. More information must be supplied about where a user's personal information (i.e., age, gender, disability, class, race, diagnosis, physical activity, income) may be shard or sold to, including private or public organisations or government services (161). The parents in this study described feeling invaded by the prospect of engaging with a DMH tool and sharing their personal information. To accommodate for this, DMH tools should be designed with “opt in” options for demographic data collection, to allow a participant to remain as unidentifiable as they feel comfortable with (16). End users should be offered an option to choose how much of their demographics they disclose, given the implications of categorical, binary Westernised diagnoses and the wider implications across gender, race, class and other social factors (161). This will have to be balanced with the necessary minimum requirement for details to provide an accurate assessment or screening. Additionally, we recommend further data governance education is offered to young people and their families, to raise awareness of the potential dual use of their data both in the present and in the future. This could be done as part of community and government campaigns, which participants in this study had said helped regarding reducing mental health stigma.

### 5.4 DMH tools as a deidentified support option

Participant preferences for what a DMH tool could do included improving access to mental health care, prevention and intervention, and providing personalised or tailored mental health support. For effective uptake, DMH tools need to be simple in their language and interface. The European Psychiatric Association recommends that DMH tools should be designed to require minimal technical specifications to access them (i.e., devices and technical support); include education resources for both parents, young people and their care team; a measure to assess the end user's digital skills; and provide guidelines or simple instructions on how to use the DMH tool (162). This would reduce the burden or exclusion of parents who do not have the technological skill set, language, or education levels to engage in a tool.

The findings that participants would separate their identities or levels of online performativity poses a challenge when designing DMH tools that are being trained on digital data or require a degree of accurate information to provide an appropriate psychological assessment. The young people in this study frequently used online personas to manage what they disclosed and to whom but also identified a preference for personalised integrated online support networks, highlighting a tension for DMH tool development between maintaining privacy whilst designing DMH tools that can be tailored and personalised. Future DMH tools could leverage this by allowing users to create pseudonymous or user-controlled personas while selectively sharing data with nominated contacts, balancing confidentiality with meaningful support. Recent HCI research has illustrated the usefulness of collecting rich qualitative narratives from participants that are then implemented with AI-powered systems to generate vignettes that illustrate emotional experiences in a tangible and scaffolded approach (163, 164). They may include the end user's behaviour patterns, moods and cognitions, and involve their nominated support people using pseudonymous or user-controlled personas that are relatable to the young person. The use of pseudonymous or user-controlled personas could may help reduce, but does not eliminate, the risk of a young person's mental health status being identified or misused (e.g., in future employment, migration, or insurance settings). For example the use of emoticons and emojis can be useful for tracking a young person's mood over time (165). Studies have found chatbots that use avatars that are non-judgemental to be more approachable and engaging (166). The use of pseudonymous or user-controlled personas could increase privacy and trust in these emerging technologies. Furthermore, the use of digital personas could address future privacy issues such as if a young person's mental health status was later reidentified or misused (e.g., in future employment, migration, or insurance settings). The Human Computer Interaction and User Experience fields frequently use personas to create imagined or speculative users to assist as fictional representations of real user profiles (167–170) but there is limited research to date on personas being created by end users to represent a deidentified version of themselves as part of signing up to a DMH app or tool. Fully anonymous platforms can provide young people the space to express themselves freely without fear of judgement and can be increasingly useful when used in tandem with offline supporters who are culturally safe (126). Pseudonymous profiles or user-controlled personas could represent one potential design approach, however, these should not be assumed to provide de-identification, as underlying data may remain linkable to an individual.

Personal identity can be fluid, particularly for young people who are constantly evolving and learning about who they are, within themselves and their social context. Consideration of this fluidity is needed in the design of DMH tools, to ensure young end users are not categorised or contrasted but are instead able to express themselves with a sense of agency and self-determination. Whilst parents were more hesitant about combining their personal information and records, they prioritised seeking mental health care for their child, and discussed a streamlined, centralised app that collected and held sensitive mental health updates. One design direction raised by these findings is an integrated DMH tools that incorporate multiple members of a young person's care network (i.e., parent, teacher, clinician, general practitioner). However this must be approached cautiously given participants' strong and repeated concerns about surveillance and unwanted disclosures. It will be important for DMH tool designers and developers to ensure that these tools do not play on a parent's vulnerabilities or eagerness to protect and care for their child. Similarly, there is risk that an integrated network could reproduce the family or school surveillance dynamics discussed in section 5.2. Instead parents can be empowered through the use of DMH tools [e.g., online peer support for parents to reduce the risk of mental health problems for young people (83) or digital voice assistants to help parents support their child's mental health (171)]. DMH tools that train parents in how they can effectively support their children whilst considering sociocultural factors, including community stigma or previous healthcare discrimination, and provide instructions to communities on how to use the technology (11) would help reduce the health disparities experienced by parents with intersecting social identities (172).

Furthermore, DMH tools that consider socioecological factors to understand initial engagement for parents with parenting programs should consider intrapersonal and interpersonal factors, as well as organisations (who is facilitating the program) and community factors (i.e., lower social cohesion or greater financial strain), along with public policy factors (i.e., societal values around parenting) (173). Prior HCI and mental health research demonstrates that digital mental health tools can be both personalised and scalable when designed with attention to users' structural and sociocultural contexts (174). The contradictions expressed by the young people in our study of wanting both control over disclosure as well as connected support underscore the need for flexible, user-centred designs. Of course this poses a challenge as the young people in this study expressed preferences for not sharing or disclosing mental health information with parents, raising important considerations for how DMH technologies might facilitate selective disclosure while avoiding unintended parental surveillance. Recognising and analysing such tensions can guide researchers and designers in creating tools that accommodate diverse youth priorities and allow for honest self-expression while enabling effective, personalised care. The table below (Table 2) captures the key recommendations from the findings of this study.

**Table 2:** Key recommendations from the study's findings'.

Design focus	Key recommendation	Intended outcome/rationale
Privacy and Identity	Enable individual users to create pseudonymous or user-controlled personas when engaging with DMH tools.	Supports honest and safe self-expression while enhancing trust and privacy in digital contexts.
Trust and Transparency	Provide simple explanations of data sharing and storage when users download or engage with DMH tools; use “opt-in” demographic data collection and allow users to control disclosure levels.	Increases perceived safety and agency, improving engagement and data-sharing comfort
Data Literacy and Governance	Offer data governance education to young people and families.	Builds awareness of data use and long-term implications, enhancing digital mental health literacy.
Parental Engagement	Design tools that consider intrapersonal and interpersonal socioecological factors influencing parents' engagement.	Acknowledges context-specific barriers and motivations shaping parental participation.
Parental Literacy and Support	Provide psychoeducation and peer-support options to reduce stigma and increase awareness.	Improves parental mental health literacy and supports families managing youth mental health challenges.
Social Identities and Inclusion	Apply a social identities framework to identify barriers such as mistrust, discrimination, and cultural mismatch.	Prevents deficit framings of “non-engagement” and recognizes how inequality and lived experience shape technology use.
Peer and Community Support	Establish peer-to-peer support networks for young people and caregivers.	Creates collective spaces for shared learning, reducing isolation and enhancing trust in digital interventions.
Integrated Care Systems	Include teachers, counsellors, and clinicians within DMH tool ecosystems with age appropriate revocable consent and clearly defined crisis-override protocols.	Fosters a coordinated, holistic support network across educational and clinical settings.
Educator Capacity Building	Provide mental health literacy and training to teachers.	Equips educators to identify and support young people's emerging mental health needs.
Accessibility and Usability	Design DMH tools with simple language and streamlined interfaces.	Ensures accessibility and comprehension across diverse literacy and digital skill levels.
System Integration	Develop centralized apps that securely collect and share mental health updates among caregivers, teachers, and clinicians (with consent).	Supports continuity of care and reduces fragmentation in service delivery.

## 6 Limitations and future research

One central limitation was that there was an imbalance in ages of young people between the two groups, with the regional group (mean = 16.3 years) being younger on average than the urban group (mean = 21.3 years). This likely influenced participant ideas around the schooling system, parental influence and autonomy given those in the urban group had more participants (80%) who were over 18 (considered an adult in Australia). Although the study incorporated a culturally and geographically diverse sample of young people and parents from both regional and metropolitan areas, the relatively small sample size constraints the extent to which meaningful comparisons can be drawn between these groups or broader generalizations that can be made. Participants were recruited through multicultural mental health organisations, with a preference for individuals who had some level of mental health literacy due to the project's time constraints. Consequently, the study may not have captured the perspectives of families with limited mental health literacy. The qualitative design of the study aimed to capture the depth and nuance of participants' perspectives rather than to achieve representativeness. Accordingly, interpretations of culturally or contextually shaped experiences were made cautiously, either when participants themselves explicitly linked their perspectives to these factors or when interpretations were substantiated by existing literature. We also note that intersectionality was applied as an interpretive lens for identifying patterns across the sample, rath than through case-by-case analysis of how multiple identities intersected within individual participants. A more granular case-level intersectional analysis is an important direction for future work. Future research would also benefit from involving young people and parents of young people in the design process from the start, to form their own research questions and frame the activities in a way that resonated with urban and regional perspectives and nuances, as well as analyze the data and review the final manuscript, which could then empower participants and the proposed design recommendations could truly reflect their needs and perspectives.

## 7 Conclusion

This paper compares the experiences of parents and young people living in different locations in seeking mental health support and their preferences for engaging with DMH tools. Barriers such as structural injustices, stigma and discrimination shaped parents' and young people's social identity which in turn was associated with their attitudes toward help seeking. Both regional and urban parents and young people were aware of the use and possible re-use of their mental health data, with both generations taking steps to keep their data deidentified or separate. The school setting plays a critical role in early mental health support and the integration of DMH tools must carefully balance the benefits with the concerns regarding surveillance, autonomy and trust. Our findings suggest that designing DMH tools that incorporate digital personas that are deidentified and confidential may promote safe and honest sharing and increase trust. Lastly, our findings point to the value of a digital integrated support network that allows a young person's caregivers, teachers, peers and additional support or care team to access their mental health updates, which may encourage community engagement and holistic support for young people engaging with DMH tools.

## Data Availability

The raw data supporting the conclusions of this article will be made available by the authors, without undue reservation.
